# Analysis of key flavor compounds in commercial cherry wines by HS-SPME-GC-MS, HS-GC-IMS, and sensory evaluation

**DOI:** 10.3389/fnut.2025.1631912

**Published:** 2025-10-03

**Authors:** Jiawei Qi, Jie Li, Zihan Liu, Xuemei Yang, Tingting Qi, Guoqin Wei, Juanxia Yang, Cheng Liu

**Affiliations:** ^1^Shandong Institute of Pomology, Tai'an, Shandong, China; ^2^College of Food Science and Engineering, Shandong Agricultural University, Tai'an, Shandong, China

**Keywords:** cherry wine, volatile flavor compounds, sensory evaluation, characteristic flavor compounds, HS-GC-IMS, HS-SPME-GC-MS

## Abstract

To investigate the characteristic flavor compounds and their differences in commercially available cherry wines, this study employed headspace solid-phase microextraction combined with gas chromatography–mass spectrometry (HS-SPME-GC-MS), headspace gas chromatography–ion mobility spectrometry (HS-GC-IMS), and sensory evaluation techniques to comprehensively analyze the volatile flavor compounds and sensory characteristics of 11 commercial cherry wines. The results showed that HS-SPME-GC-MS and HS-GC-IMS identified 74 and 49 volatile compounds, respectively. The combined use of both techniques detected a total of 101 compounds, identifying 15 additional key compounds compared to a single method, significantly improving the comprehensiveness of flavor characterization. Through odor activity value (OAV), relative odor activity value (ROAV), and variable importance in projection (VIP), 28 key flavor compounds were screened. Among them, esters (such as ethyl acetate, ethyl hexanoate, and ethyl octanoate) and alcohols (such as phenethyl alcohol) were the main contributors to the fruity and floral aroma of cherry wines, while benzaldehyde and 3-furfural were significantly associated with woody characteristics. Sensory evaluation indicated that sample C_10_ received the highest overall score, exhibiting prominent floral and fruity notes. Partial least squares regression (PLSR) analysis further confirmed that the floral and fruity attributes were strongly positively correlated with compounds such as linalool and α-terpineol, while the woody attribute was closely associated with benzaldehyde. Based on volatile compound fingerprinting and principal component analysis (PCA), samples C_5_, C_9_, and C_10_ exhibited similar flavor profiles with notable floral and fruity characteristics, whereas C_11_ displayed a unique woody character due to its high benzaldehyde content. This study provides a theoretical basis and data support for improving the flavor quality and differentiated quality control of cherry wine.

## 1 Introduction

Cherries are highly favored by consumers for their bright colors and rich nutritional components (1). Notably, cherries are rich in bioactive compounds, such as flavonoids, polyphenols, and anthocyanins. These substances possess excellent antioxidant capabilities (2). However, freshly harvested cherries have a relatively short shelf life due to their high water and sugar content. To extend their shelf life and enhance economic viability, cherries are frequently processed into juice or fruit wine.

The flavor of fruit wines is of utmost importance in determining their sensory quality and is a key element that impacts consumer preference (40). Extensive studies have been conducted on the flavor compounds in fruit wines. Wattanakul et al. (3) discovered that ethyl esters and acetate constituted the key flavor groups in the process of mango wine fermentation, while fatty acid methyl esters showed positive correlations with volatile acids, sugars, aldehydes, higher alcohols, and ketones. Li et al. (4) found that isoamyl acetate, β-damascenone, 2,3-butanediol, and phenyl ethanol were the main contributors to sweetness in dry red wine, while berry flavor characteristics were formed by geranylacetone, ethyl 2-methylbutyrate, and ethyl isobutyrate.

In cherry wine, volatile flavor compounds including alcohols, aldehydes, ketones, esters, and monoterpenes engage in intricate interactions with one another. These interactions are responsible for generating the unique and characteristic flavors that define cherry wine. Through their interactions, these diverse compounds combine to create a complex flavor profile, with each component contributing to the overall sensory experience, making the flavor of cherry wine distinct and recognizable (5). Nevertheless, most cherry wines on the market have less distinct characteristic aromas, weaker fruit and wine aromas, and suboptimal taste profiles (6).

A diverse range of chromatographic techniques, including gas chromatography (GC), gas chromatography-olfactory determination (GC-O) (7), and gas chromatography-mass spectrometry (GC-MS) (8) have been utilized for detecting the aroma compounds in fruit wines. Currently, numerous investigations have been conducted on analyzing the aroma compounds in cherry wines. Solid phase microextraction (SPME) and headspace sampler (HS) combined with gas chromatography (GC/FID) were utilized to determine the flavor compounds of Marasca cherry wine produced under varying fermentation conditions. It was found that ethyl butyrate, butyl acetate, and hexyl acetate had the highest content and imparted a fruit aroma to cherry wine (9). Xiao et al. (10) analyzed the aroma composition and aroma active ingredients of three different-priced kiraschins with HS-SPME-GC-MS and noticed that kiraschins with different prices exhibited diverse characteristics in aroma combination and aroma properties. Niu et al. (11) employed GC-O and GC-MS to conduct an in-depth study on the ester aroma compounds in cherry wine. Their research findings indicate that several esters, including ethyl butyrate, ethyl acetate, ethyl caprylate, ethyl isovalerate, ethyl decanoate, methyl salicylate and hexyl acetate, play a crucial role in endowing cherry wines with their characteristic sweetness and fruity aroma. However, there is a paucity of reports on determining the characteristic flavor compounds in cherry wines through the integration of various coupled chromatographic techniques and sensory analysis.

Although researchers have analyzed the aroma components of cherry wine using instruments such as GC-MS and conducted preliminary evaluations of its quality through sensory assessments, current studies still exhibit significant limitations: the detection methods are relatively singular, there is a lack of systematic analysis of differences in characteristic flavor compounds among different products, and the relationship between flavor substances and sensory attributes has not yet been established. To further reveal the diversity and characteristics of the flavor of commercially available cherry wines, this study selected 11 cherry wines from different brands as samples and employed a combination of HS-SPME-GC-MS and HS-GC-IMS techniques to comprehensively capture their volatile flavor compounds. By integrating chemical analysis with sensory evaluation and applying multivariate statistical methods, this research aims to differentiate the flavor profiles of cherry wines from various brands, identify key discriminatory compounds, and establish correlations between flavor components and sensory properties. The study seeks to elucidate the product characteristics of commercially available cherry wines from a flavoromics perspective, providing data support and theoretical reference for understanding their flavor composition and market differentiation.

## 2 Materials and methods

### 2.1 Material information

Ten different types of cherry wines (C_1_-C_6_,C_8_-C_11_) with high consumer ratings and market share were purchased from the Chinese e-commerce platform JD.com. C_7_ sample was obtained through homemade production. The main process is as follows: Cherries were rinsed, stemmed, and crushed. The sugar content was adjusted to 20%, followed by yeast inoculation. Fermentation was carried out at 20°C. After completion, the fermented mixture was filtered and stored in a sealed container at 4°C, with details provided in Table 1.

**Table 1:** Basic information of 11 cherry wines.

**ID**	**Name of wines**	**Ingredients**
C_1_	Cherry wine	Cherry, sugar, potassium metabisulfite, potassium sorbate
C_2_	Jingzhou cherry wine	Cherry, sugar, fructosolic acid, potassium sorbate
C_3_	Libido cherry wine	Cherry, sugar, edible alcohol, potassium sorbate
C_4_	Vantone cherry wine	Cherry, sugar, honey, sorbic acid, sulfur dioxide
C_5_	Kiplenburg cherry wine	Cherry, sugar, lactic acid, potassium pyrosulfite
C_6_	Liqueur cherry wine	Cherry, sugar, potassium sorbate
C_7_	Brew cherry wine	Cherry, potassium pyrosulfite, potassium sorbate
C_8_	Qi Fu cherry wine	Water, glutinous rice, cherry, wine koji, potassium sorbate
C_9_	Darling Cat cherry wine	Cherry, potassium sorbate, potassium pyrosulfite
C_10_	Sangria cherry wine	Cherry, potassium pyrosulfite, potassium sorbate
C_11_	Chateau Chaffey cherry wine	Cherry, vodka, sugar, flavoring, potassium sorbate

C_7_: The specific production process was as follows: cherries were cleaned, stemmed, and crushed. The sugar content was adjusted to 20%, followed by yeast inoculation. Fermentation was carried out at 20 °C. After completion, the fermented mixture was filtered and stored in a sealed container at 4 °C.

### 2.2 Physiochemical parameters analysis

In accordance with the Chinese national standard GB/T 15038-2006, the reducing sugars, total sugars, total soluble solids, total acids, alcohol content, and pH in cherry wines were measured.

### 2.3 HS-GC-IMS analysis

The HS-GC–IMS analysis method was referenced from the approach of Xie et al. (12) with modifications. Briefly, 2.0 g of cherry wine was transferred into a 20-mL headspace vial, and duplicate samples were obtained. An MXT-5 chromatographic column (15 m × 0.53 mm, 1 μm) was utilized to desorb the volatile compounds at 60 °C. The MXT-5 column excels at separating compounds based on their volatility (boiling point). This provides sufficient separation for the subsequent IMS dimension while enabling faster analysis speeds and improved robustness. An automatic headspace injection technique was used, with an injection volume of 100 μL. The cherry wine was placed at 65 °C for 10 min while rotating at 500 rpm. The temperature of injection needle was maintained at 85 °C. Nitrogen served as the carrier gas, with a total analysis time period of 40 min. Initially, the gas flow rate was set at 2 mL/min and held constant for 2 min, then increased to 10 mL/min over the following 8 min, and further raised to 90 mL/min over the following 30 min. Additionally, nitrogen at a flow rate of 150 mL/min was utilized as the drift gas. Each group of samples was analyzed in duplicate.

### 2.4 HS-SPME-GC-MS analysis

The procedure was referenced from the method of Niu et al. (11) with modifications for analyzing the volatile compounds in cherry wine using HS–SPME–GC–MS. The specific operational steps are as follows: a 20 mL sample of cherry wine was transferred to a container with a volume of 50 mL, followed by adding 4 g of NaCl and 80 μL of 2-octanol (0.411 g/L). The container was then sealed with a Teflon lid. The SPME fiber (DVB/C-WR/PDMS) head was inserted into the container and allowed to adsorb volatile compounds at 50 °C for 45 min, with continuous stirring using a magnetic stirrer bar. The analysis was conducted with GC-MS (GCMS-TQ8040 NX). A VF-WAX capillary column (30 m × 0.25 mm × 0.55 μm) was employed, with helium as the carrier gas at a flow rate of 1.2 mL/min. The primary reason for using a polar column (VF-Wax) in HS-SPME-GC-MS is that the flavor compounds analyzed by GC-MS are typically polar, thereby maximizing the resolution and sensitivity of the analysis. Additionally, the retention indices and mass spectra obtained from the detection can be directly compared with data from large commercial databases (e.g., NIST), significantly enhancing the accuracy and reliability of compound identification. The specific operational procedure is as follows: the injection port maintained at 260°C and the injection was conducted in splitless mode. The temperature program was as follows: the initial temperature was set at 45°C and held for 1 min, then increased at a rate of 4 °C/min to 80 °C and maintained for 2 min. It was then raised at a rate of 5 °C/min to 230°C and held for 10 min. The ion source temperature remained constant at 230 °C. The ionization was performed in EI mode, with an electron energy of 70 eV and a mass scanning range of 30–450 m/z. Volatile compounds were identified by matching their mass spectra against the NIST 20.0 mass spectral library. 2-Octanol is typically absent in cherry wine. It does not belong to the group of major or common alcohols, such as ethanol, isoamyl alcohol, or isobutanol, produced during the fermentation of cherry wine. 2-Octanol exhibits a well-defined peak shape and stable retention time in GC analysis, making it suitable for using as an internal standard. Quantitative analysis of volatile components was performed by comparing the peak area with that of the internal standard (2-octanol). Each group of samples was analyzed in triplicate.

### 2.5 Calculation of odor activity value and relative odor activity value

The OAV value represents the ratio of the mass concentration to the odor threshold of a volatile compound and is primarily used to assess its contribution to the cherry wine aroma (13). Typically, volatile compounds with an OAV ≥ 1 remarkably affect the flavor profile of cherry wine (14). The following formula is used for the calculation of OAV:


(1) $$\begin{array}{l} \text{OA}{\text{V}}_{\text{I}}=\;\frac{C_{i}}{T_{i}} \end{array}$$

Where C_i_ represents the compound concentration, T_i_ denotes the threshold level of compound, and OAV_i_ indicates the value of odor activity associated with the compound.

The formula for calculating ROAV is presented as follows:


(2) $$\begin{array}{l} \text{ROA}{\text{V}}_{\text{i}}=100\;\times \;\frac{OAV_{i}}{OAV_{\max}} \end{array}$$

ROAV_i_ is the relative odor activity value of a compound, while OAV_max_ refers to the maximum OAV value among all compounds in the sample.

### 2.6 Sensory analysis

The sensory evaluation panel consists of 14 trained wine tasters, comprising 6 males and 8 females, with the ages from 23 to 45 years old. All tasters panelists had prior experience in wine tasting. Additionally, to enhance the accuracy of cherry wine evaluation, the 14 panelists received a 1-week training session specifically on cherry wine tasting. The entire sensory analysis adhered to sensory ethical standards. Before the experiment commenced, every participant was comprehensively briefed on the study's objective. Subsequently, they all furnished written consent to take part in the experiment. All panelists were non-smokers and had no known medical conditions, particularly those associated with the oral and olfactory organs. The sensory evaluation of cherry wine was conducted according to the requirements of GB/T15038-2006. Fourteen panelists evaluated the samples, describing the wine based on six aroma attributes (floral, fruity, fermented, burnt, sour, and woody) and scoring each wine sample accordingly.

### 2.7 Data analysis

In this study, SPSS 26.0 software is used for one-way analysis of variance. SIMCA software is employed to conduct OPLS-DA and VIP analysis on the volatile compounds of the samples. PLSR analysis was performed with Unscramble X. For visualizing HS-GC-IMS data, interpolation software such as Reporter, Gallery Plot, and Dynamic PCA was utilized. This combination of analytical and visualization tools enabled a comprehensive and in-depth exploration of the data, providing deeper insights into the relationships and characteristics within the dataset.

## 3 Results and discussion

### 3.1 Physicochemical indicators of cherry wines

Table 2 summarizes the basic physical and chemical indices of 11 kinds of cherry wines. The cherry wine with the lowest total sugar content is C_2_ (34.93 g/L), while the one with the highest is C_11_ (318.53 g/L). The reason why the total sugar content of C_11_ is greater than that of other wines may be the addition of some sugar to enhance the taste. The initial sugar level directly determines the potential alcohol content, which in turn influences the aroma profile via the alcohol degree (15). Ethanol is an excellent organic solvent that enhances the solubility of many aroma compounds—such as higher alcohols and esters (e.g., ethyl acetate, ethyl hexanoate)—in the wine, allowing them to remain more stable within the matrix rather than volatilizing. This contributes to a richer and more complex aromatic profile (16). Acidity determines the pH environment of cherry wine, and pH acts as a critical condition for many chemical reactions (17). A lower pH value (i.e., higher acidity) favors the progression of esterification, thereby promoting the formation and accumulation of fruit-aroma ester compounds—such as ethyl acetate and ethyl hexanoate (18). Excessively high acidity may bring an off—flavor to cherry wine, while insufficient acidity may lead to a lack of flavor. The total acid content of C_1_ is the highest (6.95 g/L), and the significantly lower total acid content of C_11_ (1.92 g/L) compared to other wines may be related to its production process. The 11 kinds of cherry wines show significant differences in various physical and chemical indices.

**Table 2:** Basic physicochemical indicators of 11 cherry wines.

**ID**	**Total sugars (g/L)**	**Reducing sugar (g/L)**	**Total acid (g/L)**	**pH**	**Alcohol content (%,V/V)**
C_1_	105.53 ± 1.33^g^	103.73 ± 1.63^f^	6.95 ± 0.75^a^	4.32 ± 0.01^a^	8.38 ± 0.10^e^
C_2_	34.93 ± 4.73^a^	19.83 ± 10.26^b^	4.89 ± 0.26^de^	3.27 ± 0.01^h^	10.10 ± 0.13^c^
C_3_	121.43 ± 1.65^f^	94.73 ± 0.91^g^	3.74 ± 0.26^fg^	4.06 ± 0.01^b^	8.67 ± 0.13^d^
C_4_	131.73 ± 0.65^e^	110.93 ± 3.35^ef^	4.98 ± 0.20^de^	3.74 ± 0.02^e^	6.86 ± 0.06^h^
C_5_	89.03 ± 1.50^i^	69.03 ± 0.75^i^	6.59 ± 0.28^c^	3.67 ± 0.01^f^	7.67 ± 0.06^g^
C_6_	131.93 ± 0.12^e^	115.97 ± 0.12^de^	6.34 ± 0.43^c^	3.86 ± 0.00^d^	8.00 ± 0.06^f^
C_7_	162.63 ± 0.60^c^	130.40 ± 5.03^c^	5.60 ± 0.45^b^	3.75 ± 0.01^e^	6.50 ± 0.07^i^
C_8_	150.63 ± 3.50^d^	120.57 ± 1.17^d^	3.62 ± 0.38^g^	3.56 ± 0.00^g^	10.45 ± 0.06^b^
C_9_	94.03 ± 2.23^h^	87.10 ± 0.66^gh^	4.55 ± 0.29^ef^	4.04 ± 0.00^c^	8.58 ± 0.05^d^
C_10_	89.97 ± 0.35^hi^	78.93 ± 4.81^h^	5.53 ± 0.41^d^	3.57 ± 0.00^g^	6.90 ± 0.09^h^
C_11_	318.53 ± 1.45^b^	263.20 ± 2.50^a^	1.92 ± 0.38^h^	3.21 ± 0.00^i^	29.55 ± 0.07^a^

Different letters represent significant differences in the 11 groups of samples (*P* < 0.05); Data are presented as mean ± standard deviation (*n* = 3).

### 3.2 HS-GC-IMS results of the cherry wines

The volatile flavor compounds characterized through HS-GC-IMS are listed in Table 3. A total of 49 signal peaks were characterized in 11 cherry wine samples, such as 1 organic acid, 3 aldehydes, 4 ketones, 8 alcohols, and 14 esters. Additionally, due to database limitations, 15 compounds remained unidentified. Additionally, 15 compounds remained unidentified due to database limitations. Certain flavor compounds are present as proton-bound dimers and monomers, likely resulting from variations in volatile compound concentrations, which can generate multiple signals, including dimers and even trimers (19).

**Table 3:** The VOCs identified by HS-GC-IMS in cherry wines.

**NO**.	**Compounds**	**Odor threshold (μg/L)**	**MW**	**RI**	**Rt**	**Dt**	**Relative content (%)**										
							**C**_1_	**C**_2_	**C**_3_	**C**_4_	**C**_5_	**C**_6_	**C**_7_	**C**_8_	**C**_9_	**C**_10_	**C**_11_
1	Ethyl octanoate-M	5 (36)	172.3	1,440.6	832.836	1.47287	0.24	0.08	0.08	0.09	0.27	0.05	0.08	0.03	0.25	0.23	0.04
2	Ethyl octanoate-D	5 (36)	172.3	1,439.8	831.527	2.03022	0.63	0.15	0.09	0.09	0.49	0.06	0.08	0.06	0.66	0.44	0.05
3	Ethyl hexanoate	5 (36)	144.2	1,240.2	506.45	1.79914	5.62	0.95	1.45	2.34	8.76	7.86	1.60	0.46	10.10	13.18	0.49
4	Ethyl crotonate	UN	114.1	1,141.4	361.85	1.54666	1.31	0.29	0.23	2.14	1.13	2.50	1.20	1.15	0.96	0.65	0.76
5	Isoamyl acetate	3000 (36)	130.2	1,130.4	348.246	1.74977	3.28	0.32	0.18	8.33	7.30	4.57	5.86	7.26	9.32	12.29	12.85
6	Ethyl 3-methylbutanoate	450 (36)	130.2	1,074.5	289.295	1.65731	0.39	0.27	0.87	1.23	0.41	0.53	0.04	1.07	0.53	0.31	0.05
7	Ethyl butanoate	20 (36)	116.2	1,046.8	265.56	1.56509	2.32	13.13	2.07	1.33	3.22	2.55	0.17	7.21	4.61	5.95	6.11
8	Ethyl acetate	7500 (36)	88.1	900.3	176.217	1.34136	10.88	14.76	19.36	12.86	11.85	10.84	14.34	11.39	9.05	9.80	10.28
9	Ethyl propanoate	1500 (36)	102.1	966.5	209.998	1.46045	0.50	0.07	0.11	0.28	0.20	0.36	0.18	0.04	0.32	0.56	0.01
10	Ethyl isobutyrate	900 (36)	116.2	976.1	215.406	1.56725	0.37	0.03	0.09	1.05	1.70	0.53	0.02	0.05	0.35	0.68	0.01
11	Methyl acetate	50 (36)	74.1	845.6	152.499	1.19891	3.65	2.17	3.97	4.23	1.47	2.31	3.04	2.49	1.61	2.03	1.78
12	3-methylbutyl butanoate	160 (37)	158.2	1,270.6	560.3	1.94024	0.16	4.04	0.22	0.17	0.22	0.19	0.24	2.13	0.16	0.34	9.81
13	Ethyl heptanoate	90 (37)	158.2	1,347.8	681.223	1.92063	0.02	0.13	0.04	0.02	0.03	0.02	0.03	0.03	0.02	0.02	0.53
14	Ethyl 2-methylbutanoate	250 (37)	130.2	1,057.3	274.339	1.656	0.22	0.17	0.47	0.31	0.18	0.13	0.03	0.06	0.48	0.11	0.04
15	1-Octen-3-ol	UN	128.2	1,441	833.593	1.59065	0.80	0.37	0.37	0.38	1.05	0.24	0.25	0.10	1.03	0.92	0.16
16	(Z)-3-hexenol	8000 (37)	100.2	1,369.9	714.588	1.54663	16.79	7.48	6.32	4.01	5.47	16.90	8.59	16.45	9.00	2.71	2.64
17	1-Pentanol-M	5000 (37)	88.1	1,214	464.321	1.51132	19.67	6.81	14.07	23.04	21.50	16.46	21.18	4.32	17.45	17.49	5.80
18	1-Pentanol-D	5000 (37)	88.1	1,212.4	461.837	1.80315	2.23	0.60	0.97	2.67	2.64	1.84	2.52	0.30	1.98	2.07	0.28
19	1,8-cineole	120 (37)	154.3	1,212.8	462.458	1.32561	3.05	9.26	9.83	3.51	3.04	2.91	4.46	4.74	2.88	2.12	5.76
20	3-pentanol	8500 (37)	88.1	1,136.8	356.107	1.41432	3.00	1.47	0.68	3.41	3.79	2.30	3.78	4.49	3.05	2.52	4.15
21	Butan-2-ol	20000 (37)	74.1	1,043.6	262.981	1.31992	0.93	2.86	1.95	0.76	0.95	0.59	1.41	0.84	0.60	0.43	0.86
22	3-Heptanol	250 (37)	116.2	1,293.5	604.693	1.64831	0.22	0.34	0.12	0.10	0.18	0.12	0.25	1.00	0.16	0.21	0.20
23	Octanal	40 (37)	128.2	1,301.4	616.151	1.40967	0.38	0.17	0.06	0.21	0.10	0.19	0.17	0.09	0.14	0.09	0.13
24	Benzaldehyde	2000 (37)	106.1	1,509.5	966.99	1.4777	0.16	0.21	1.85	0.24	0.41	0.10	0.15	9.10	3.90	5.09	9.28
25	Furfural	770 (38)	96.1	1,469.5	886.591	1.3392	0.32	0.15	0.88	0.36	0.12	0.57	0.16	0.19	0.15	0.09	0.50
26	4-Methyl-2-pentanone	5000 (38)	100.2	1,024.2	247.676	1.48277	0.66	0.11	0.14	1.75	0.60	1.34	0.76	0.08	0.23	0.53	0.40
27	Hexan-2-one	10000 (38)	100.2	1,077.1	291.581	1.50624	0.28	0.15	0.30	0.78	0.19	0.56	0.06	0.10	0.12	0.14	0.06
28	2-Octanone	250 (36)	128.2	1,291.9	601.374	1.33397	0.22	0.08	0.13	0.17	0.14	0.19	3.49	0.86	0.13	0.09	0.03
29	3-hydroxy-2-butanone	5 (36)	88.1	1,264.4	548.954	1.32499	0.94	0.63	0.90	1.52	1.23	0.95	1.76	0.33	0.91	1.03	0.44
30	Acetone	200 (36)	58.1	830.5	146.514	1.11941	0.23	0.41	1.09	0.31	0.55	0.75	0.96	0.39	0.33	0.36	0.43
31	Alpha-terpinolene-M	UN	136.2	1,296.6	609.74	1.20933	3.12	6.26	6.61	4.23	2.70	4.25	7.43	6.35	2.67	2.06	2.80
32	Alpha-terpinolene-D	UN	136.2	1,286.1	589.856	1.72858	0.05	0.16	0.10	0.06	0.07	0.05	0.11	0.12	0.08	0.09	0.27
33	Alpha-Pinene	400 (36)	136.2	1,007	234.872	1.21752	0.98	0.58	1.02	1.90	0.91	1.34	1.32	0.62	0.59	0.87	2.60
34	Propionic acid	11200 (36)	74.1	1,510.3	968.511	1.27651	0.28	1.48	4.04	1.00	1.05	0.80	0.24	2.33	1.90	1.88	3.01
35	1	-	0	1,439.7	831.284	1.79614	0.36	0.13	0.09	0.07	0.15	0.10	0.07	0.05	0.19	0.08	0.04
36	2	-	0	1,468.8	885.404	1.45304	0.22	0.16	0.19	0.09	0.11	0.36	0.08	0.13	0.12	0.10	0.06
37	3	-	0	1,240.1	506.341	1.63794	2.31	0.50	0.57	2.28	2.66	1.73	1.53	0.25	2.00	1.84	0.32
38	4	-	0	1,043.4	262.817	1.39134	0.88	0.31	0.26	0.22	0.37	0.31	0.96	0.12	0.48	0.23	0.04
39	5	-	0	1,025.6	248.764	1.34545	0.39	0.19	0.17	0.62	0.66	0.54	0.59	0.11	0.40	0.47	0.49
40	6	-	0	1,021.3	245.51	1.61952	0.44	0.06	0.04	1.60	1.21	0.90	0.61	0.03	0.27	0.61	5.85
41	7	-	0	965.5	209.461	1.27742	1.33	1.90	1.90	0.80	0.95	0.97	0.99	0.90	1.27	1.23	0.68
42	8	-	0	976.1	215.405	1.32782	0.46	0.38	0.85	0.69	0.60	0.53	0.17	0.45	0.57	0.49	0.03
43	9	-	0	1,149.4	372.116	1.3426	0.60	0.29	0.40	0.44	0.27	0.75	0.38	0.20	0.26	0.14	0.14
44	10	-	0	986.4	221.383	1.28584	0.80	0.83	0.68	0.24	0.18	0.42	1.23	0.33	0.84	0.58	0.24
45	11	-	0	1,054.4	271.857	1.40814	0.12	0.32	0.18	0.14	0.06	0.29	0.07	0.06	0.09	0.05	0.07
46	12	-	0	1,056.1	273.297	1.45936	0.09	0.09	0.14	0.17	0.07	0.18	0.04	0.05	0.04	0.05	0.04
47	13	-	0	1,045.3	264.363	1.45479	0.08	0.13	0.17	0.31	0.09	0.19	0.17	0.05	0.04	0.04	0.04
48	14	-	0	1,067.7	283.305	1.32136	0.10	0.82	0.15	0.10	0.13	0.13	0.10	0.33	0.14	0.24	0.34
49	15	-	0	1,273	564.91	1.52115	0.18	4.31	0.12	0.25	0.18	0.11	0.18	1.95	0.17	0.19	1.83

-M: Represents the monomer ion peak ([M+H]^+^); -D: Represents the dimer ion peak ([2M+H]^+^).

**Figure 1:**
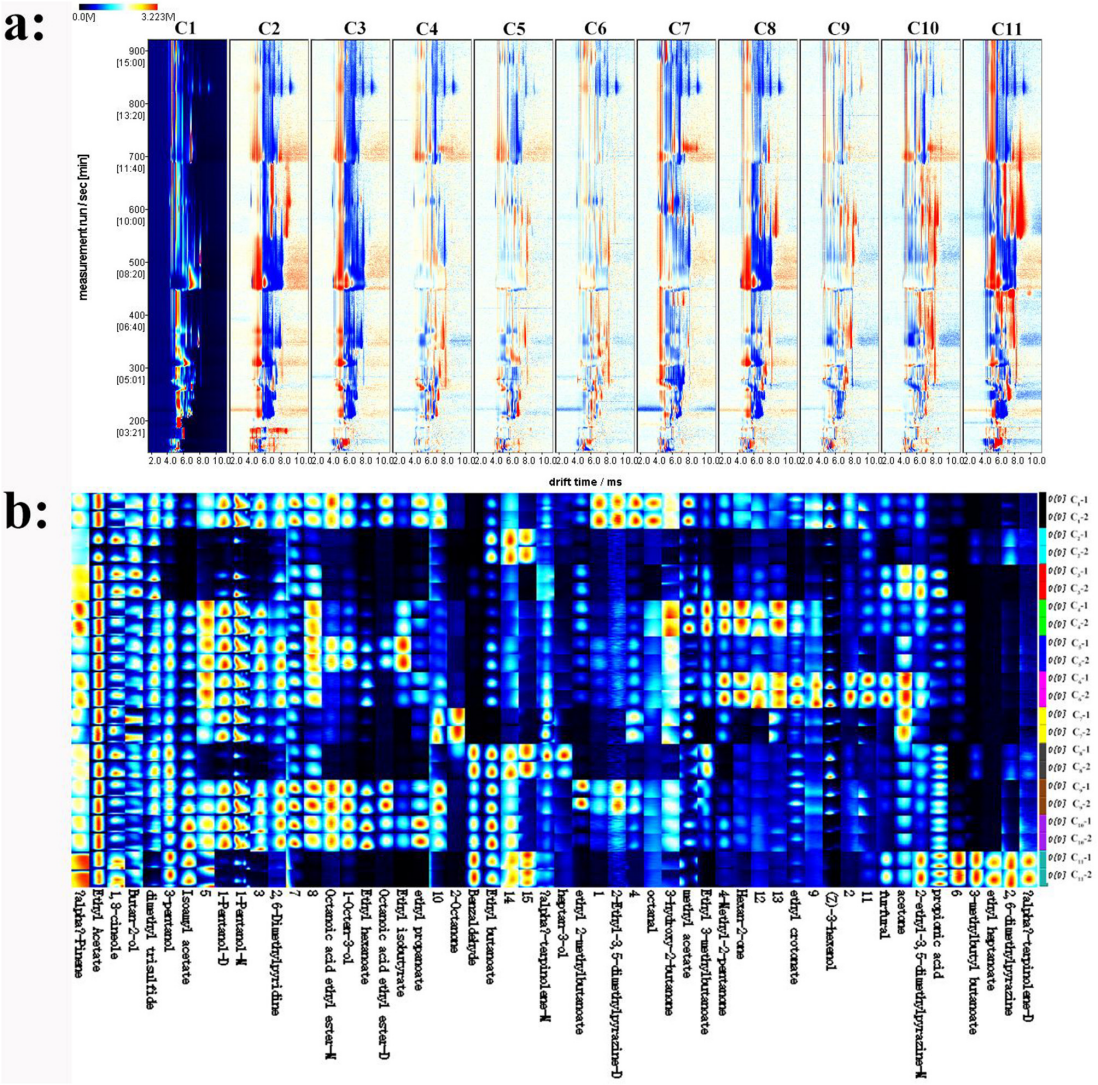
Analyzing volatile compounds in cherry wines with HS-GC-IMS. **(a)** Comparative difference map of volatile compounds. **(b)** Fingerprint of volatile compounds.

In order to assess the differences among samples, the sample C_1_ spectrum was utilized as a benchmark. Subsequently, the remaining samples' spectra were withdrawn from that of sample C_1_. This approach allowed for a clear visualization and quantification of the variances between the samples, facilitating a more in—depth analysis of their unique characteristics. A white subtracted background indicates consistent flavor compounds; red means a greater concentration than the reference, and blue represents a lower one. As presented in Figure 1a, which illustrates the comparison of volatile compound content in different cherry wine samples with C_1_ as the reference, the main compound content in C_4_, C_5_, C_6_, C_9_, and C_10_ samples were similar to those in C_1_.

To clearly highlight the specific differences in compounds among cherry wines, each peak was chosen for fingerprint comparison (Figure 1b). The volatile compounds in different cherry wines were qualitatively characterized through fingerprint analysis. In Figure 1b, each row represents all the signal peaks selected for a specific sample, while each column corresponds to the signal peaks of the same volatile organic compounds (VOCs) across different cherry wines. The figure employs color coding to indicate the relative content of VOCs, providing a clear and intuitive visualization of their distribution and abundance. Brighter colors signify higher concentrations (20). This figure offers a comprehensive overview of the volatile compound profiles in each sample, highlighting the differences in VOC composition among them. It enables a detailed examination of the unique volatile compound profiles of individual samples and emphasizes the variations between them. Notably, α-pinene, ethyl acetate, 1,8-cineole, and 2-butanol are present in relatively high concentrations in the cherry wines. α-Pinene and 1,8-cineole contribute woody and resinous aromas, while ethyl acetate and 2-butanol impart a fruity aroma. These compounds may serve as key characteristic volatiles in cherry wine, significantly influencing its overall flavor profile. Among the samples, C_1_ contains the highest levels of octanal, 3-hydroxy-2-butanone, and methyl acetate. C_2_ exhibites the highest concentration of ethyl butyrate, which provides a pronounced fruity aroma. However, apart from ethyl butyrate and two unidentified compounds (designated as 14 and 15), the types and numbers of the remaining volatile compounds in C_2_ were substantially lower than in other samples. Additionally, 2-octanone was abundant in C_7_, imparting a distinct natural woody and herbal aroma.

### 3.3 Results from HS-SPME-GC-MS analysis of the cherry wines

To obtain a deeper awareness of the volatile organic compounds (VOCs) in cherry wines, the HS-SPME-GC-MS technique was employed. This analysis successfully identified 74 VOCs, such as 16 alcohols, 25 esters, 8 ketones, 6 phenols, 8 acids, 7 aldehydes, and 4 monoterpenoids (as detailed in Table 4). Marked differences were observed in the types and concentrations of volatile compounds among the 11 cherry wine varieties. In terms of total volatile compound concentration, C_1_ and C_10_ exhibited the highest levels, measuring 396.46 mg/L and 819.05 mg/L, respectively. In contrast, the total concentration in the remaining cherry wines ranged between 60 and 200 mg/L, with most samples clustering around 100 mg/L. Variations in volatile compound composition were also evident among the samples. Notably, C_7_ had the greatest number of volatile compounds (42 types) but had a relatively lower total concentration. In contrast, C_10_ had the fewest volatile compounds (27 types) but exhibited a significantly higher total concentration.

**Table 4:** HS-SPME-GC-MS analysis: VOCs, thresholds and odor traits in cherry wines.

**NO**.	**Compounds**	**Odor threshold (μg/L)**	**Odor**	**Content (**μ**g/L)**										
				**C**_1_	**C**_2_	**C**_3_	**C**_4_	**C**_5_	**C**_6_	**C**_7_	**C**_8_	**C**_9_	**C**_10_	**C**_11_
1	Ethyl acetate	7,500 (36)	Pineapple	42,435.86	42,435.84	28,746.33	37,998.16	50,599.38	11048.68	9170.02	9,377.90	10,543.24	8,450.28	25,640.87
2	Ethyl butanoate	44 (36)	Fruity	142.57	264.04	ND	ND	ND	ND	ND	122.28	ND	ND	ND
3	Pentyl acetate	30 (36)	Banana	65.70	ND	ND	38.86	ND	ND	ND	ND	ND	ND	ND
4	Ethyl hexanoate	14 (36)	Fennel, fruit	4,146.75	179.11	1,299.84	88.52	976.52	1147.52	1342.95	2,318.51	2,440.48	1,769.89	551.86
5	Ethyl lactate	60,000 (36)	Sweet, greasy	1,859.64	588.66	4,721.25	231.16	2,786.72	9000.28	1106.43	11,833.33	9,073.56	663.20	1,533.93
6	Ethyl furan-2-carboxylate	200 (37)	Sweet, fruity	ND	ND	293.03	ND	ND	ND	ND	671.60	93.80	ND	ND
7	Ethyl pentanoate	450 (38)	Sweet, fruity	136.35	ND	ND	ND	ND	137.39	0.00	ND	ND	ND	ND
8	Ethyl heptanoate	220 (38)	Fruity, pineapple	306.72	236.06	ND	ND	ND	ND	ND	ND	ND	ND	785.14
9	Ethyl octanoate	5 (36)	Floral scent	805.35	538.48	330.14	843.22	950.37	174.69	662.64	377.04	2,470.54	2,314.96	ND
10	Ethyl decanoate	200 (36)	Fruity	262.34	277.80	ND	ND	377.00	ND	265.25	193.81	621.81	937.01	ND
11	Ethyl benzoate	553 (11)	Fruity	1,530.71	4,480.55	10,913.63	ND	ND	ND	ND	4,735.73	6,180.20	ND	ND
12	Diethyl butanedioate	300,000 (11)	Fruity	ND	ND	703.67	0.00	2,825.12	11539.72	1005.71	633.12	7,140.61	4,911.47	ND
13	Ethyl phenylacetate	73 (11)	Apple, fruity	189.62	72.38	96.42	213.35	154.51	ND	ND	360.10	619.12	460.72	ND
14	Hexyl acetate	600 (11)	Fruity	148.57	258.81	78.23	171.32	368.55	77.47	190.04	46.39	88.44	974.72	ND
15	2-Phenylethyl acetate	250 (37)	Fruity	52.12	ND	ND	ND	672.26	680.41	385.51	ND	ND	0.00	ND
16	Benzyl acetate	364 (37)	The scent of roses	ND	ND	ND	ND	ND	ND	ND	ND	ND	1,886.26	ND
17	Methyl salicylate	20.2 (11)	Spices, herbs	ND	ND	ND	ND	ND	ND	237.71	ND	ND	ND	7,979.63
18	Ethyl dodecanoate	3,500 (11)	Pine tree	ND	ND	184.02	ND	ND	ND	ND	ND	ND	ND	4,287.76
19	Ethyl 3-phenylpropanoate	1.6 (38)	Floral scent	ND	ND	ND	ND	ND	94.74	165.00	ND	ND	ND	ND
20	Ethyl hexadecanoate	UN	Fruity, creamy and fermented	73.06	253.25	521.94	67.08	ND	140.09	ND	ND	ND	ND	ND
21	Dimethyl phthalate	UN	Fruity, creamy	ND	ND	ND	831.64	ND	ND	522.86	ND	ND	ND	ND
22	Isoamyl acetate	30 (11)	Tasteless	53.09	295.73	ND	56.69	1,587.02	ND	ND	1,478.90	1,832.18	6,216.62	5,537.85
23	Isoamyl butyrate	160 (36)	Banana, fruity	ND	346.82	ND	ND	ND	ND	ND	548.85	ND	1,551.67	6,475.42
24	(2E,4E)-Ethyl hexa-2,4-dienoate	UN	Green apple, fruity	ND	1,170.10	459.46	622.54	ND	ND	266.38	262.85	1,2383.79	444.89	ND
25	Isopropyl myristate	UN	Fruity, sweet, green, pineapple	ND	218.67	ND	ND	156.12	605.12	235.65	1,035.49	459.07	291.17	ND
26	1-Butanol	150,000 (36)	Pungency	ND	ND	ND	ND	ND	ND	ND	ND	439.16	ND	ND
27	1-Propanol, 2-methyl-	40,000 (36)	Malt	132.22	ND	606.18	179.52	2,484.93	597.33	264.97	ND	ND	ND	29,050.59
28	1-Butanol, 3-methyl-	30,000 (36)	Malt, Whisky	336,467.38	1,148.55	8,929.87	4,607.39	21,044.14	22062.66	9612.33	1,904.72	4,6460.29	24,893.18	75,686.47
29	1-Hexanol	2,500 (37)	Grass,green	ND	ND	ND	540.62	ND	ND	5542.02	534.11	6,932.69	4,200.75	7,866.15
30	2-Methyl-2-[2-(2,6,6-trimethylcyclohex-2-enyl)ethyl][1,3]dioxolane	UN	Honey	ND	ND	ND	ND	ND	ND	ND	ND	ND	ND	1,915.52
31	1-Hexanol, 2-ethyl-	2,000 (37)	Citrus fragrance	ND	ND	ND	101.26	ND	ND	1,262.83	ND	ND	108.88	ND
32	2,3-Butanediol	450 (37)	Floral scent	338.97	ND	ND	ND	ND	1,935.29	357.50	ND	ND	ND	ND
33	1-Octanol	25.2 (36)	Floral, fruity	ND	ND	ND	37.14	ND	ND	245.95	ND	889.81	142.70	ND
34	Linalool	50 (37)	Citrus fragrance	308.13	295.23	77.99	80.96	147.53	2,896.48	979.92	83.63	ND	7,609.44	ND
35	3-Furanmethanol	180 (37)	Floral scent	84.28	ND	442.84	ND	ND	482.45	357.32	244.77	ND	ND	ND
36	1-Decanol	600 (36)	Flowers, roses	ND	ND	ND	ND	ND	ND	ND	ND	346.92	ND	ND
37	1-Nonanol	10 (36)	Rosin, wood	ND	ND	ND	ND	ND	ND	193.92	ND	ND	ND	ND
38	.alpha.-Terpineol	400 (36)	Green lemon	92.09	66.27	ND	572.74	2,668.92	3,595.39	378.48	129.28	493.21	9,170.74	1,956.41
39	Citronellol	100 (39)	Floral scent	ND	ND	ND	ND	ND	893.48	ND	ND	ND	457.09	ND
40	Benzyl alcohol	200,000 (39)	Flowers, roses	1,174.87	306.04	3,077.73	4,036.00	263.21	1,084.76	135.77	1,241.03	1,510.12	500.77	ND
41	Phenylethyl Alcohol	1,000 (37)	Earthy, fatty	593.12	743.42	1,056.02	18,206.54	2,826.35	8,665.37	3,098.82	482.80	8,165.17	7,195.09	ND
42	3-Hexen-1-ol, (E)-	1,000 (37)	Floral scent	ND	ND	ND	91.66	ND	ND	ND	417.39	ND	214.39	ND
43	1-Hexadecanol	400 (36)	Fat, earthy, honey	ND	ND	ND	ND	ND	ND	157.20	ND	ND	ND	ND
44	1-Dodecanol	40,000 (36)	Pungency	ND	ND	ND	ND	ND	ND	0.00	153.06	109.13	162.33	ND
45	2-Heptanone	1,000 (36)	Cheese, fruit, coconut	ND	ND	ND	ND	ND	ND	141.62	ND	ND	ND	ND
46	2-Octanone	50 (37)	Floral, herbal, fruity	ND	ND	ND	ND	152.42	216.35	208.57	ND	ND	289.94	ND
47	Acetoin	5,000 (38)	Creamy dairy products, milk	102.69	ND	ND	33.45	ND	263.72	2,815.81	ND	262.38	ND	ND
48	2-Propanone, 1-hydroxy-	25,000 (37)	Caramel	ND	ND	ND	0.00	ND	240.04	170.36	ND	0.00	ND	ND
49	2-Nonanone	100 (38)	Fruity	ND	ND	ND	42.08	ND	ND	146.96	ND	0.00	ND	ND
50	Alpha-Ionone	2.6 (38)	Fruity	ND	1,593.65	ND	ND	ND	ND	ND	ND	1,405.28	ND	ND
51	Isophorone	100 (38)	Costus	ND	280.81	ND	ND	ND	ND	ND	ND	ND	1,981.76	ND
52	Acetophenone	3,000 (37)	Flora	ND	ND	430.58	ND	ND	ND	ND	ND	ND	ND	ND
53	Phenol, 4-ethyl-2-methoxy-	1,100 (39)	Fruity	70.23	ND	ND	ND	ND	ND	ND	ND	ND	ND	ND
54	Phenol, 3-ethyl-	5 (39)	Musty smell	ND	ND	ND	963.57	ND	ND	ND	ND	ND	ND	ND
55	Phenol, 4-ethyl-	405 (36)	Smoky taste	279.33	ND	258.34	ND	ND	332.80	ND	ND	ND	ND	ND
56	2,4-Di-tert-butylphenol	200 (36)	UN	1,606.10	2,824.82	ND	4,933.46	ND	ND	ND	ND	3,441.26	1,246.30	ND
57	4-tert-Butylbenzenethiol	UN	UN	ND	294.55	1,705.21	ND	184.28	ND	ND	184.20	ND	ND	12,899.09
58	Phenol	2,500 (36)	Rubber	ND	ND	ND	ND	ND	ND	175.62	0.00	150.84	88.19	ND
59	Acetic acid	1,000 (36)	Aroma of vinegar	696.15	742.60	1,444.32	1,596.75	1,381.83	13,506.47	9,055.81	2,383.97	3,346.93	2,480.17	ND
60	Butanoic acid	4,000 (36)	Sour cheese	ND	ND	ND	ND	ND	ND	ND	ND	481.03	0.00	ND
61	Hexanoic acid	420 (38)	Unpleasant odor, metallic taste	126.78	ND	ND	158.27	672.34	1,321.32	2,357.22	ND	8,356.10	6,154.61	ND
62	Octanoic acid	500 (36)	Greasy smell	525.82	777.94	221.78	4,373.20	116.85	2,364.58	4,397.57	262.09	733.24	1,6014.15	7,510.83
63	n-Decanoic acid	1,000 (38)	Soapy, waxy, fruity	91.46	ND	ND	1,484.65	2,532.36	937.27	712.99	170.06	17,880.05	1,1575.47	5,488.12
64	Sorbic Acid	200 (39)	Tasteless	103.48	702.26	3,213.57	368.53	ND	1,468.86	724.04	244.09	1,767.93	993.11	4,369.81
65	Dodecanoic acid	1,000 (39)	Fatty, waxy	ND	ND	ND	ND	ND	ND	195.69	407.70	583.24	ND	ND
66	Benzoic acid	85 (11)	Balsam	ND	ND	ND	413.50	ND	52.53	ND	ND	ND	ND	ND
67	3-Furaldehyde	770 (11)	Sweet and fragrant	340.62	139.83	3,614.67	141.73	169.11	3,297.99	366.41	976.28	1,183.51	1,299.96	2,1846.03
68	Decanal	7 (39)	Waxy, fat, citrus and orange peel	ND	ND	ND	ND	ND	ND	508.38	ND	ND	2,610.74	ND
69	Benzaldehyde	2,000 (36)	Fruity, almond notes	1,043.82	1,775.82	6,295.36	297.89	3,985.76	1,155.44	457.80	37,782.71	23,875.25	18,605.86	576,526.44
70	Benzaldehyde diethylacetal	UN	Bitter almond	ND	ND	ND	ND	ND	ND	ND	227.06	ND	ND	5,093.42
71	Benzaldehyde, 3,4-dimethyl-	UN	Almond flavor	78.59	ND	ND	ND	ND	ND	ND	ND	ND	ND	ND
72	5-Hydroxymethylfurfural	500,000 (11)	Butter, musty, caramel	ND	ND	744.03	ND	ND	ND	1,088.59	441.95	511.74	142.65	11977.33
73	2,5-Furandicarboxaldehyde	UN	UN	ND	ND	ND	ND	ND	ND	ND	ND	ND	ND	4066.45

UN, Unable to obtain relevant information; ND, Indicates that the alarm is not detected.

Among all the volatile compounds detected, esters were the most predominant in both quantity and content. These compounds are produced via the esterification of fatty acids with alcohols (21). Studies have shown that esters play a critical role in defining the aroma of fruit wines and are key contributors to their fruity taste and sweetness (22). Ethyl esters, in particular, are essential aroma components in fruit wines, with their concentrations influenced by factors including yeast strains, sugar content, and fermentation temperature. These compounds significantly enhance the overall quality of fruit wines, as most exhibit ripe and fruity aromas, contributing to the “floral” and “fruity” sensory characteristics (23). Ethyl acetate, ethyl caproate, ethyl lactate, and ethyl caprylate were detected in all 11 cherry wines, with their average concentrations exceeding their respective threshold levels. Previous studies have similarly concluded that ester compounds, including ethyl butyrate, ethyl acetate, ethyl caprylate, and ethyl lactate, impart a distinctive fruit aroma and play an essential role in the volatile flavor profile of fruit wines (24). Each ester contributes specific sensory attributes: ethyl acetate typically imparts apple and banana aromas, ethyl caproate provides pineapple and strawberry aroma, ethyl lactate is associated with fruity and creamy aromas, and ethyl caprylate exhibits fruity and floral characteristics.

Higher alcohols are essential secondary metabolites generated during the fermentation process of *Saccharomyces cerevisiae*. At appropriate levels, they result in the smoothness and balance of cherry wines (25). However, the types and content of alcohols varied significantly among the 11 cherry wine samples. C_2_, C_3_, and C_11_ contained the fewest detected alcohols. Isoamyl alcohol, phenyl ethanol, and benzyl alcohol were present in all samples except C_11_. Phenylethanol and isoamyl alcohol, known for their floral and grassy aromas, are likely generated via yeast metabolism through the Ehrlich pathway (26). Additionally, certain alcohols were exclusive to specific wine samples. For instance, 1-butanol and n-decanol were detected only in C_9_, while 2-propyl-1-amyl alcohol, 1-cetanol, and 2-methyl-1-amyl alcohol were unique to C_4_. Diisobutylcarbinol was found only in C_8_, whereas 1-nonyl alcohol and 2-nonyl alcohol were exclusive to C_7_. Current studies have demonstrated that differences in raw materials for winemaking (such as cherry varieties) (27) and variations in fermentation process parameters (e.g., yeast strains, fermentation temperature, pH, etc.) (28) can significantly influence the yeast metabolic pathways, thereby altering the composition and content of higher alcohols. The differences observed among samples in this study may stem from the application of the aforementioned factors in the production processes of different commercial wines.

Acid compounds in cherry wines primarily originate during fermentation. Their concentrations are influenced by two key factors: Acid compounds in cherry wines primarily originate from cherry juice and yeast fermentation. Cherry juice contains citric acid, malic acid, and other organic acids, and yeast produces a large quantity of acids through glycolysis (the EMP pathway) during the fermentation. Furthermore, yeast may generate a small amount of acetic acid via the acetaldehyde pathway) under oxygen-deficient or stressful conditions (such as excessively high temperature or nutrient deficiency). Because of their high odor thresholds, acids are conducive minimally to the overall flavor profile of cherry wines. In this study, nine acids were characterized, with acetic acid, caprylic acid, and capric acid detected in all 11 samples. This aligns with previous research, confirming the consistent presence of these acids in cherry wines (10). Additionally, the corresponding ethyl esters of these acids were also detected, indicating active esterification of ethanol with free fatty acids. The presence of these fatty acids suggests that a robust esterification reaction occurred during fermentation, playing a key role in the chemical transformation of the wine—making process. This reaction also significantly influences the flavor and aroma characteristics of cherry wines. It is noteworthy that benzoic acid was detected in samples C4 and C6 of this study. Although cherry raw materials may inherently contain benzoic acid and its precursors (29), in alcoholic products, the detection of free benzoic acid is generally regarded as an indicator of the addition of preservatives such as sodium benzoate. This may be a process measure adopted by producers to ensure product stability.

Seven aldehydes were identified in cherry wines, with benzaldehyde being present in all samples. Notably, C_8_ and C_11_ demonstrated markedly higher benzaldehyde concentrations in comparison to the other samples, surpassing its odor threshold. This suggests that benzaldehyde could have a more pronounced influence on the aroma profile of C_8_ and C_11_. At appropriate concentrations, benzaldehyde imparts fruity, sweet, nutty, and caramel-like notes (30). Although aldehydes generally have low OAV values, they can enhance woody aroma intensity while reducing fruity aroma perception (31). The sensory analysis of C_11_ revealed it has a strong oak aroma, further supporting the role of benzaldehyde and other aldehydes in shaping the peculiar flavor profile of cherry wines.

Apart from the previously mentioned volatile compounds, 9 types of ketones and 6 types of phenols were also detected in the cherry wines. Although these compounds present in relatively low concentrations, their low sensory thresholds allow them to significantly influence the overall flavor profile. Research findings indicate that ketones have the ability to augment the flavor complexity in cherry wines. They play a pivotal role in orchestrating the release of various aromas (32).

### 3.4 Sensory evaluation

**Figure 2:**
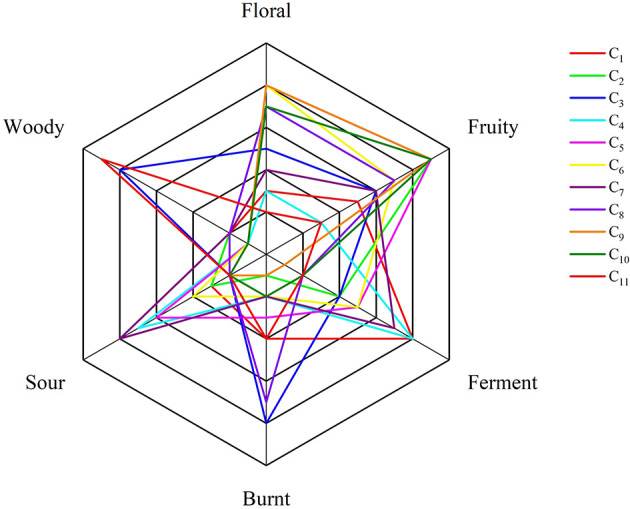
Aroma scores for cherry wines.

The sensory evaluation and aroma scoring results for cherry wine, assessed by 14 trained panelists, are illustrated in Figure 2. The sensory profiles of the cherry wines encompass a diverse range of aroma attributes, including fruity, sour, woody, fermented, roasted, and floral notes. Notably, most cherry wines exhibited dominant floral and fruity characteristics, suggesting that these aroma components significantly enhance the overall aromatic appeal of cherry wine. This observation aligns with the volatile compound analysis, where esters and alcohols key contributors to floral and fruity aromas were observed in high concentrations.

### 3.5 Comparison of analysiss between HS-GC-IMS and HS-SPME-GC-MS

#### 3.5.1 Comparison of PCA analysis results

**Figure 3:**
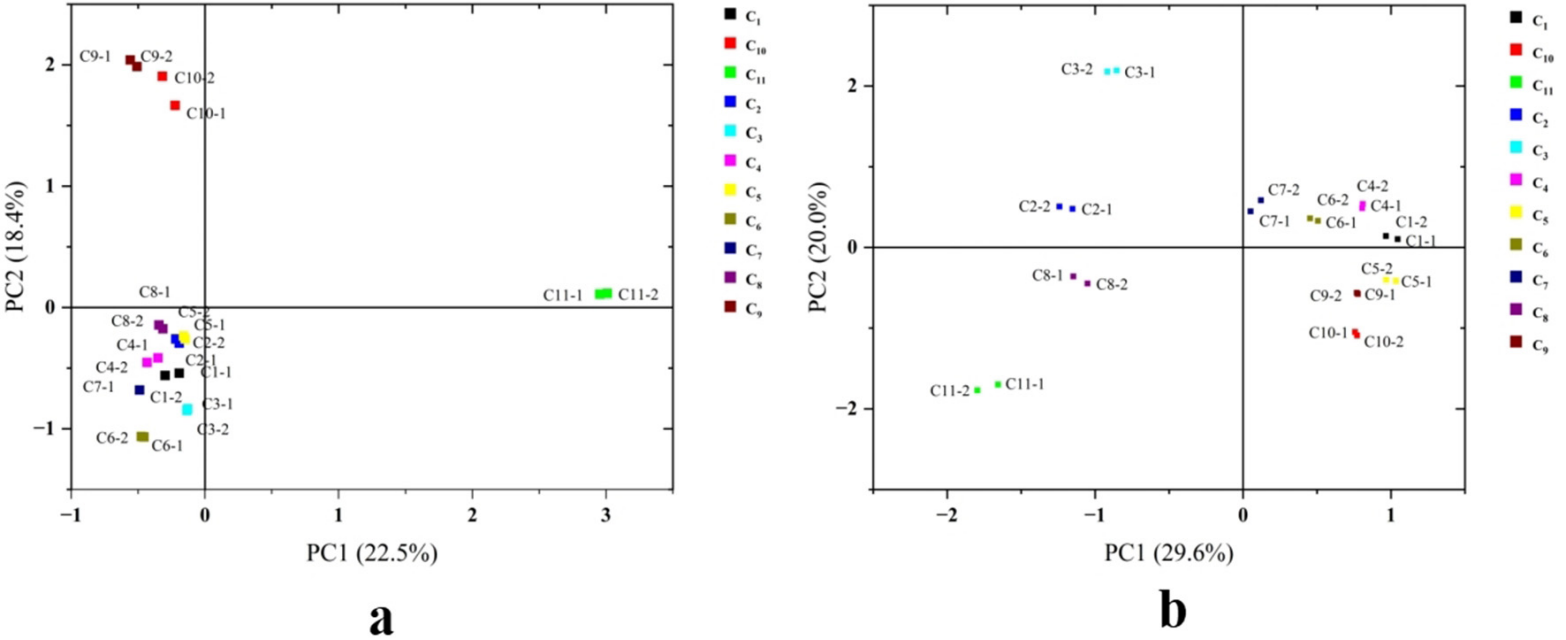
PCA analysis of cherry wines. **(a)** PCA score plot for HS-SPME-GC-MS. **(b)** PCA score plot for HS-GC-IMS.

PCA is an unsupervised multivariate statistical analysis technique that effectively captures variations in volatile components among samples. By applying PCA, researchers can assess the consistency and variability of different samples, facilitating more accurate comparisons and interpretations of similarities and differences. In PCA plots, samples with similar flavor characteristics cluster together, whereas those with distinct flavor profiles are positioned farther apart. As exhibited in Figure 3, PCA results of HS-SPME-GC-MS and HS-GC-IMS revealed certain variations. Notably, C_1_, C_4_, C_5_, C_6_, and C_7_ were closely positioned in both analytical methods, suggesting minimal differences in their volatile compound profiles. This finding indicates that these cherry wines likely share similar aroma characteristics. In contrast, C_11_ was distinctly separated from the other samples, highlighting significant differences in its volatile compound composition. This separation suggests that C_11_ possesses a unique aromatic profile, potentially contributing to distinct sensory attributes compared to the other cherry wine samples.

#### 3.5.2 A comparison of the detection results from HS-GC-IMS and HS-SPME-GC-MS

The flavor compounds identified using the HS-SPME-GC-MS and HS-GC-IMS techniques were predominantly esters and alcohols, reinforcing their key role in determining the flavor profile of cherry wines. This result aligns with previous research, confirming the substantial contribution of flavor compounds to the overall sensory characteristics of cherry wines. However, notable differences were observed between the two analytical methods. Compared to GC-MS, GC-IMS demonstrated superior sensitivity in detecting dimer volatiles, terpenes, and heterocyclic compounds. This advantage stems from the distinct detection principles of IMS and MS (33). Despite its strengths, HS-GC-IMS is an effective method in food flavor analysis, but its compound identification capability is constrained by the NIST database, making it unable to identify some compounds. Some of these unidentified compounds may have a significant impact on flavor. As a result, certain compounds remain unidentified, some of which could significantly influence the flavor profile (34). For example, in the HS-GC-IMS results (Figure 1b), unidentified compounds 3, 7, 8, 10, and 15 were present at high levels in the cherry wines, suggesting their potential importance in defining the wine's aroma. Regarding the identification of volatile components, HS-SPME-GC-MS identified 75 compounds, whereas HS-GC-IMS identified only 49 compounds. Some compounds detected by HS-GC-IMS were absent in HS-SPME-GC-MS, including ethyl propionate, ethyl isovalerate, 1-octene-3-ol, (Z)-3-hexanol, 1-pentanol-M, 1-pentanol-D, 1,8-cineole, 3-pentanol, and tertiary amyl alcohol. These differences highlight the complementary nature of the two techniques. To achieve a comprehensive characterization of volatile flavor compounds in cherry wines, a combined approach with both HS-SPME-GC-MS and HS-GC-IMS is necessary. Together, these methods provide a more complete and nuanced understanding of the wine's flavor composition.

### 3.6 The key flavor compounds of cherry wines

#### 3.6.1 OPLS-DA analysis

**Figure 4:**
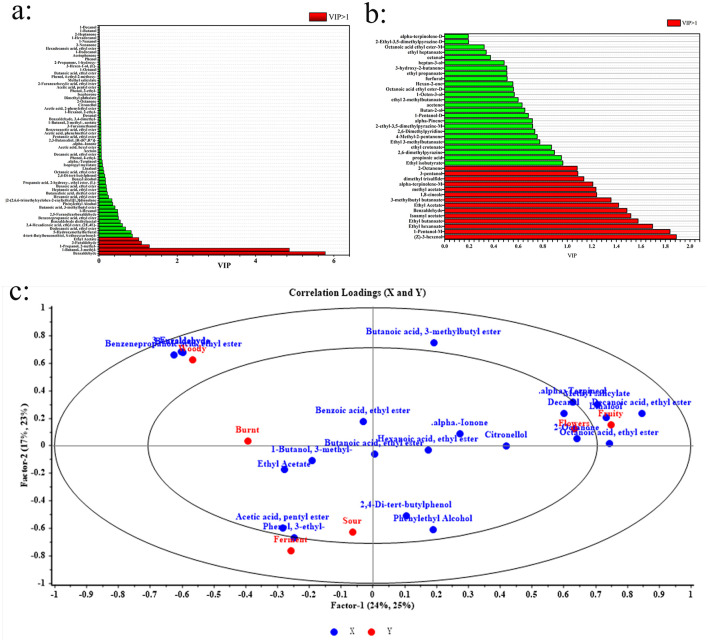
Analysis of key flavor compounds in cherry wines. **(a)** VIP rankings of HS-SPME-GC-MS. **(b)** VIP rankings of HS-GC-IMS. **(c)** PLSR analysis of characteristic flavor compounds and sensory properties of cherry wines.

To assess each VOC's contribution to the flavor profile of cherry wines, the VIP values within the OPLS-DA model were analyzed. VOCs with VIP values over 1 were considered remarkable contributors to the overall aroma and taste characteristics (35). Figures 4a, b display the VIP values of VOCs identified by HS-SPME-GC-MS and HS-GC-IMS, respectively, according to the OPLS-DA model. From the HS-SPME-GC-MS analysis, five VOCs including benzaldehyde, 1-butanol, 2-methyl, 3-furaldehyde, 3-methyl, 1-propanol, and ethyl acetate exhibited VIP values exceeding 1, indicating their major influence on the flavor profile. These compounds contribute distinct sensory attributes, with benzaldehyde imparting sweet, nutty, and fruity notes, while ethyl acetate enhances fruity and floral characteristics. In contrast, HS-GC-IMS identified 14 VOCs with VIP values over 1, emphasizing the broader range of impactful compounds detected by this method. These key contributors included (Z)-3-hexenol, 1-pentanol-M, ethyl hexanoate, ethyl butanoate, isoamyl acetate, benzaldehyde, ethyl acetate, 3-methylbutyl butanoate, 1,8-cineole, methyl acetate, alpha-terpinolene-M, dimethyl trisulfide, 3-pentanol, and 2-octanone. The presence of additional esters, alcohols, and terpenes in this list highlights the enhanced sensitivity of HS-GC-IMS in detecting flavor-relevant compounds. These findings confirm that benzaldehyde and ethyl acetate were consistently identified as significant contributors across both analytical methods, further reinforcing their importance in determining the characteristic aroma of cherry wines. The differences in detected key VOCs also underscore the necessity of combining HS-SPME-GC-MS and HS-GC-IMS to achieve a comprehensive understanding of cherry wine flavor composition.

#### 3.6.2 The determination of the OAV by HS-SPME-GC-MS and the ROAV by HS-GC-IMS

To better understand the volatile flavor compounds' contribution to the overall composition of cherry wines, the OAV of VOCs was calculated. The OAV is determined by dividing the concentration of a compound by its corresponding odor threshold. A higher OAV indicates a stronger influence of the compound on the wine's overall flavor profile. Combining HS-SPME-GC-MS with OAV analysis provides an effective method for identifying the key flavor compounds in cherry wines. As shown in Table 5, compounds with an OAV >1 included 12 esters, 6 alcohols, 4 ketones, 2 phenols, and 3 aldehydes. Specifically, the esters detected were ethyl acetate, ethyl butyrate, isoamyl acetate, ethyl heptanoate, ethyl hexanoate, ethyl octanoate, ethyl benzoate, ethyl phenylacetate, methyl salicylate, ethyl phenylpropionate, and isoamyl butyrate. Among the alcohols, n-hexanol was identified. The terpenoid compounds included linalool, α-terpineol, and citronellol, while the ketones comprised 2-octanone, α-ionone, and isophorone. The phenolic compounds detected were 3-ethylphenol and 2,4-di-tert-butylphenol, and the aldehydes included decanal and benzaldehyde. The key flavor compounds identified were consistent with previous studies (11). Isoamyl acetate and ethyl hexanoate contributed a banana-like aroma, while ethyl octanoate imparted a pineapple aroma. Ethyl acetate and ethyl butyrate enhanced the fruity and floral scent. 2-Octanone and α-ionone provided fruity characteristics, whereas n-hexanol reinforced the floral aroma. Benzaldehyde was a major contributor to the woody aroma, and monoterpenes such as citronellol, geraniol, and α-terpineol contributed floral, fruity, and citrusy nuances.

**Table 5:** VOCs (OAV>1) of the volatile compounds in cherry wines by HS-SPME-GC-MS.

**NO**	**Compounds**	**OAV**										
		**C**_1_	**C**_2_	**C**_3_	**C**_4_	**C**_5_	**C**_6_	**C**_7_	**C**_8_	**C**_9_	**C**_10_	**C**_11_
1	Ethyl acetate	5.66	5.66	3.83	5.07	6.75	1.47	1.22	1.25	1.41	1.13	3.42
2	Ethyl butanoate	3.24	6.00	-	-	-	-	-	2.78	-	-	-
3	Pentyl acetate	2.19	-	-	1.30	-	-	-	-	-	-	-
4	Ethyl hexanoate	2,764.50	119.40	866.56	59.01	651.01	765.02	895.30	1,545.67	1,626.99	1,179.93	367.91
5	Ethyl furan-2-carboxylate	-	-	1.47	-	-	-	-	3.36	0.47	-	-
6	Ethyl heptanoate	1.39	1.07	-	-	-	-	-	-	-	-	3.57
7	Ethyl octanoate	161.07	107.70	66.03	168.64	190.07	34.94	132.53	75.41	494.11	462.99	-
8	Ethyl decanoate	1.31	1.39	-	-	1.88	-	1.33	0.97	3.11	4.69	-
9	Ethyl benzoate	2.77	8.10	19.74	-	-	-	-	8.56	11.18	-	-
10	Ethyl phenylacetate	2.60	0.99	1.32	2.92	2.12	-	-	4.93	8.48	6.31	-
11	2-Phenylethyl acetate	0.21	-	-	-	2.69	2.72	1.54	-	-	-	-
12	Benzyl acetate	-	-	-	-	-	-	-	-	-	5.18	-
13	Methyl salicylate	-	-	-	-	-	-	11.77	-	-	-	395.03
14	Ethyl 3-phenylpropanoate	-	-	-	-	-	59.21	103.13	-	-	-	-
15	Isoamyl acetate	0.02	0.10	-	0.02	0.53	-	-	0.49	0.61	2.07	1.85
16	Isoamyl butyrate	-	2.17	-	-	-	-	-	3.43	-	9.70	40.47
17	1-Butanol, 3-methyl-	11.22	0.04	0.30	0.15	0.70	0.74	0.32	0.06	1.55	0.83	2.52
18	1-Hexanol	-	-	-	0.68	-	-	6.93	0.67	8.67	5.25	9.83
19	2,3-Butanediol	0.75	-	-	-	-	4.30	0.79	-	-	-	-
20	1-Octanol	-	-	-	1.47	-	-	9.76	-	35.31	5.66	-
21	3-Furanmethanol	0.47	-	2.46	-	-	2.68	1.99	1.36	-	-	-
22	1-Nonanol	-	-	-	-	-	-	19.39	-	-	-	-
23	alpha.-Terpineol	0.92	0.66	-	5.73	26.69	35.95	3.78	1.29	4.93	91.71	19.56
24	Phenylethyl Alcohol	0.59	0.74	1.06	18.21	2.83	8.67	3.10	0.48	8.17	7.20	-
25	1-Hexadecanol	-	-	-	-	-	-	0.39	-	-	-	-
26	2-Octanone	-	-	-	-	3.05	4.33	4.17	-	-	5.80	-
27	2-Nonanone	-	-	-	0.42	-	-	1.47	-	-	-	-
28	alpha.-Ionone	-	612.94	-	-	-	-	-	-	540.49	-	-
29	Phenol, 3-ethyl-	-	-	-	192.71	-	-	-	-	-	-	-
30	2,4-Di-tert-butylphenol	8.03	14.12	-	24.67	-	-	-	-	17.21	6.23	-
31	3-Furaldehyde	0.44	0.18	4.69	0.18	0.22	4.28	0.48	1.27	1.54	1.69	28.37
32	Decanal	-	-	-	-	-	-	72.63	-	-	372.96	-
33	Benzaldehyde	0.52	0.89	3.15	0.15	1.99	0.58	0.23	18.89	11.94	9.30	288.26

Compared to OAV analysis, ROAV analysis only requires data of relative concentration. Hence, ROAV analysis was performed using the volatile compounds identified through HS-GC-IMS to determine the key volatile flavor compounds in cherry wine. As exhibited in Table 6, 13 volatile organic compounds with ROAV > 1 were identified, such as ethyl hexanoate, ethyl octanoate-M, ethyl octanoate-D, ethyl butyrate, ethyl isovalerate, ethyl acetate, ethyl formate, butyl 3-methylbutyrate, 1-pentanol-M, 1,8-cineole, octyl aldehyde, 3-hydroxy-2-butanone, and acetone. In the OAV analysis, ethyl acetate, ethyl hexanoate, ethyl octanoate, and ethyl butyrate all showed OAV values over 1, confirming their significant contribution to the overall aroma. To further refine the identification of key flavor compounds, the OAV, ROAV, and VIP values were integrated. Among the esters, ethyl butyrate, ethyl acetate, amyl acetate, ethyl hexanoate, ethyl benzoate, ethyl octanoate, methyl salicylate, ethyl phenylpropionate, 3-methylbutyl butyrate, ethyl 3-methylbutyrate, and methyl acetate were included; among the alcohols, 3-methyl-1-butanol and phenylethyl alcohol and were included; among the ketone compounds, 2-octanone, α-nonanone, 3-hydroxy-2-butanone, acetone, phenol, 3-ethylphenol, and 2,4-di-tert-butylphenol were included; among the aldehyde compounds, decanal, benzaldehyde, and 3-furaldehyde were important components of the flavor of cherry wine.

**Table 6:** VOCs (ROAV>1) of the volatile compounds in cherry wines by HS-GC-IMS.

**NO**	**Compounds**	**ROAV**										
		**C**_1_	**C**_2_	**C**_3_	**C**_4_	**C**_5_	**C**_6_	**C**_7_	**C**_8_	**C**_9_	**C**_10_	**C**_11_
1	Ethyl octanoate-M	42.24	13.73	14.24	15.41	47.10	7.92	14.71	5.85	44.06	40.14	7.73
2	Ethyl octanoate-D	108.74	25.23	15.60	14.80	85.86	9.66	14.40	10.95	114.85	75.61	8.88
3	Ethyl hexanoate	976.01	164.81	252.15	406.00	1,520.88	1,364.87	278.56	80.51	1,754.23	2,288.71	84.35
4	Isoamyl acetate	0.95	0.09	0.05	2.41	2.11	1.32	1.70	2.10	2.70	3.56	3.72
5	Ethyl butanoate	100.78	569.82	89.90	57.91	139.81	110.61	7.34	312.78	199.95	258.08	265.18
6	Ethyl Acetate	1.26	1.71	2.24	1.49	1.37	1.26	1.66	1.32	1.05	1.13	1.19
7	Methyl acetate	63.29	37.73	68.84	73.52	25.50	40.02	52.80	43.16	27.93	35.25	30.92
8	3-Methylbutyl butanoate	0.88	21.94	1.20	0.94	1.18	1.05	1.28	11.54	0.88	1.84	53.24
9	1-Pentanol-M	3.41	1.18	2.44	4.00	3.73	2.86	3.68	0.75	3.03	3.04	1.01
10	1,8-Cineole	22.03	66.96	71.12	25.41	21.99	21.06	32.30	34.30	20.83	15.30	41.66
11	Octanal	8.21	3.62	1.28	4.52	2.23	4.08	3.68	1.89	3.08	1.87	2.84
12	3-Hydroxy-2-butanone	163.41	108.71	157.06	264.55	213.38	164.54	305.30	57.36	157.97	178.32	76.41
13	Acetone	1.01	1.80	4.73	1.35	2.39	3.26	4.15	1.71	1.45	1.54	1.87

#### 3.6.3 Characteristic flavor compounds correlated with sensory properties

To further investigate the rrelationship between characteristic flavor compounds and sensory characteristics, PLSR analysis was performed. In this analysis, VOCs with OAV > 1 were designated as the X variables, while sensory characteristics were set as the Y variables. The ellipses in the PLSR plot represent explained variances of 50% and 100%, respectively. If most variables fall within these ellipses, it indicates that the PLSR model effectively explains their relationships. As shown in Figure 4c, the majority of key flavor compounds exhibited a positive correlation with floral and fruity aromas. Specifically, “fruity” and “floral” characteristics were strongly associated with linalool, α-terpinol, isophorone, and ethyl caprylate. The “woody” aroma showed a strong correlation with 3-furfural and benzaldehyde, while the “scorched” aroma was closely linked to 1,8-cineole. Although these individual compounds may not independently produce a distinct sensory aroma, their interactions with other volatile compounds in cherry wine likely contribute to the formation of its characteristic flavor profile. Overall, PLSR analysis further validated the relationship between key flavor compounds and sensory properties, providing deeper insights into the flavor composition of cherry wine.

## 4 Conclusion

This work comprehensively identified the volatile flavor compounds in cherry wine using HS-SPME-GC-MS and HS-GC-IMS techniques. A total of 101 volatile compounds were identified, with esters and alcohols being the predominant contributors to the overall flavor profile. Among them, ethyl acetate, ethyl caprylate, and hexyl acetate were critical compounds accountable for the fruity aroma, while 3-furfural and benzaldehyde contributed to the woody notes. The integration of OAV and ROAV analyses enabled the identification of 28 primary flavor compounds, further validated through sensory evaluation. Notably, cherry wine samples C5, C9, and C10 exhibited the most pronounced floral and fruity characteristics. The results of this work are of great significance for clarifying the characteristic flavor substances of cherry wines, promoting the improvement of cherry wine quality, and accelerating product innovation.

## Data Availability

The original contributions presented in the study are included in the article/supplementary material, further inquiries can be directed to the corresponding author.
